# The Maize ABA Receptors ZmPYL8, 9, and 12 Facilitate Plant Drought Resistance

**DOI:** 10.3389/fpls.2018.00422

**Published:** 2018-04-04

**Authors:** Zhenghua He, Junwei Zhong, Xiaopeng Sun, Bingcai Wang, William Terzaghi, Mingqiu Dai

**Affiliations:** ^1^National Key Laboratory of Crop Genetic Improvement, Huazhong Agricultural University, Wuhan, China; ^2^Hubei Key Laboratory of Food Crop Germplasm and Genetic Improvement, Food Crops Institute, Hubei Academy of Agricultural Sciences, Wuhan, China; ^3^Department of Biology, Wilkes University, Wilkes-Barre, PA, United States

**Keywords:** ABA receptors, drought resistance, natural variation, *Zea mays*, ABA response

## Abstract

Drought is one of the major abiotic stresses affecting world agriculture. Breeding drought-resistant crops is one of the most important challenges for plant biologists. *PYR1/PYL/RCARs*, which encode the abscisic acid (ABA) receptors, play pivotal roles in ABA signaling, but how these genes function in crop drought response remains largely unknown. Here we identified 13 *PYL* family members in maize (*ZmPYL1-13*). Changes in expression of these genes under different stresses indicated that *ZmPYLs* played important roles in responding to multiple abiotic stresses. Transgenic analyses of *ZmPYL genes* in Arabidopsis showed that overexpression of *ZmPYL3*, *ZmPYL9, ZmPYL10,* and *ZmPYL13* significantly enhanced the sensitivity of transgenic plants to ABA. Additionally, transgenic lines overexpressing *ZmPYL8*, *ZmPYL9,* and *ZmPYL12* were more resistant to drought. Accumulation of proline and enhanced expression of drought-related marker genes in transgenic lines further confirmed the positive roles of *ZmPYL* genes in plant drought resistance. Association analyses with a panel of 368 maize inbred lines identified natural variants in *ZmPYL8* and *ZmPYL12* that were significantly associated with maize drought resistance. Our results deepen the knowledge of the function of maize *PYL* genes in responses to abiotic stresses, and the natural variants identified in *ZmPYL* genes may serve as potential molecular markers for breeding drought-resistant maize cultivars.

## Introduction

Due to their sessile lifestyle, plants cannot escape from environmental stresses which include biotic and abiotic stresses. Drought is one of the major abiotic stresses that negatively affect plant growth and development. Plants have evolved sophisticated mechanisms to respond to and survive drought. Generally, plants have two major mechanisms for drought resistance, drought avoidance and drought tolerance (Hu and Xiong, 2014). Drought avoidance includes increasing cuticular wax and abscisic acid (ABA) content, controlling relative water content and water potential, along with leaf rolling and stomatal aperture control. General criteria for drought tolerance are content of osmolytes such as proline and sugar, and membrane system stability (Hu and Xiong, 2014).

Abscisic acid is a phytohormone that plays critical roles in plant stress response. Under drought stress, plants increase the production of ABA, which initiates stomatal closure and thereby reduces transpirational water loss, thus helping the plant avoid drought stress (Kim et al., 2010). Plants can perceive ABA via ABA receptors, the regulatory components of the ABA receptor (RCAR) or PYRABACTIN RESISTANCE 1 (PYR1)/PYR1-like protein (PYL) family of START proteins (Ma et al., 2009; Park et al., 2009). The clade A PP2C phosphatases and SnRK2 kinases are also important in mediating ABA signaling (Miyazono et al., 2009; Fujita et al., 2011; Joshi-Saha et al., 2011; Antoni et al., 2012; Nakashima et al., 2012, 2014; Rushton et al., 2012; Roychoudhury et al., 2013). A number of studies revealed the important roles of SnRK2 kinases and clade A PP2C phosphatases in regulation of plant drought resistance. Mutation of *SnRK2.6*/*OST1* caused constitutive stomatal opening and water loss (Mustilli et al., 2002). Triple mutants of *snrk2.2*/*3*/*6* lost the ability to respond to ABA and were hypersensitive to drought (Fujii and Zhu, 2009). Recent study has revealed that SnRK2.6 regulates the ubiquitin E3 ligase activity of RZFP34/CHYR1, a positive regulator of plant drought resistance (Ding et al., 2015). In Arabidopsis, the HAI PP2Cs function in drought responses by regulating the accumulation of osmoregulatory solutes such as proline (Bhaskara et al., 2012). *ZmPP2C-A10*, encoding a clade A PP2C phosphatase, negatively regulates maize drought response (Xiang et al., 2017).

There are 14 Arabidopsis genes encoding PYR/PYL/RCAR ABA receptors (Li et al., 2013). Recently, these Arabidopsis *PYR*/*PYL* genes have been extensively studied and reported to be important for plant drought responses. By using an ABA inducible promoter, researchers have revealed that overexpressing *PYL9* upon stress induction greatly enhanced drought resistance and drought-induced leaf senescence in transgenic Arabidopsis and rice plants (Zhao et al., 2016). Overexpression of *PYL5* also enhanced plant drought tolerance (Santiago et al., 2009). The A194T site mutant of *PYL4* formed stable complex with PP2CA in the absence of ABA, and 35S:*PYL4* (A194T) plants showed dramatically enhanced tolerance to drought and dehydration (Pizzio et al., 2013). In guard cells, PYL/RCAR ABA receptors interact with PP2C phosphatases, thus releasing active SnRK2 kinase to activate the SLAC1 channel which leads to stomatal closure by reducing guard cell turgor (Lee et al., 2013). This shows the involvement of PYL/RCAR ABA receptors in plant drought avoidance. In maize, a previous study reported the expression profiles of *ZmPYL* genes in response to ABA and dehydration stress (Fan et al., 2016). However, the function of *ZmPYL* genes in regulation of maize drought tolerance remains elusive.

Association analysis based on linkage disequilibrium (LD) is widely used for dissecting complex traits of crops (Wang and Qin, 2017). By using genome wide association analysis (GWAS), a number of loci involved in regulation of complex plant traits, such as flowering time, oil synthesis, salt tolerance, were indentified recently (Huang et al., 2011; Meyer et al., 2016; Fang et al., 2017). Maize is a model plant for genetic studies due to its huge genetic diversity among various accessions (Strable and Scanlon, 2009). Due to the fast LD decay, researchers can detect trait-locus associations in maize at single gene resolution. For example, recent studies used GWAS to detect two drought-tolerant genes, *ZmNAC111* and *ZmVPP1*, from a maize population consisting of 368 accessions (Mao et al., 2015; Wang et al., 2016). Based on the same maize population, we and another group identified two drought-resistance genes, *ZmPP2C-A10* and *ZmDREB2.7*, and their natural variations through candidate gene association analyses (Liu et al., 2013; Xiang et al., 2017). Although the function of PYL/RCAR ABA receptors in regulation of drought resistance has been revealed in several plant species, how the natural variations in *PYL*/*RCAR* genes are associated with plant drought responses remains essentially unknown.

In this study, we identified and cloned the maize *PYL* family genes. Expression analyses showed that *ZmPYL* genes responded to multiple abiotic stresses including ABA, drought and salt. Transgenic analyses revealed the roles of several *ZmPYLs* in regulation of plant ABA and drought responses. Association analysis with a large maize association population identified several favorable alleles of two *ZmPYL* genes, *ZmPYL8* and *ZmPYL12*, for maize drought resistance.

## Materials and Methods

### Plant Material

Maize B73 seeds were used for gene cloning and expression analysis. The *Arabidopsis* ecotype Col-0 was used as the wild-type. The pRCS2(Bar)-*ZmPYLs* plasmids were introduced into *Agrobacterium tumefaciens* strain GV3101 and then transformed into Arabidopsis Col-0 ecotype using the floral dip method (Clough and Bent, 1998). Seeds of transformed Arabidopsis were selected on MS plates containing the appropriate antibiotics. Homozygous lines of T4 generations with one copy of inserted transgene were used for further analysis.

### Plant Growth Conditions and Treatments

All maize seedlings were grown under 16 h light: 8 h dark photoperiod at 28°C. For drought treatment, maize seedlings were germinated and grown in soil under normal watering conditions until the three-leaf stage. Plants were then transferred onto filter paper and dried at 28°C. Shoot and root samples were harvested after 0, 1, 3, 6, 12, 24 h, respectively. For ABA and salt treatments, maize seeds were sown and grown in Hoagland’s solution until the three-leaf stage. Then seedling roots were soaked in Hoagland’s solution containing various concentrations of ABA (0, 1, 10, 50, 100, and 150 μM) for 3 h. For salt treatments, plant roots were immersed in Hoagland’s solution containing 150 mM NaCl and sampled after 0, 1, 2, 3, and 4 days. Upon harvesting the samples were immediately frozen in liquid nitrogen for RNA extraction.

*Arabidopsis* seeds of wild-type and independent transgenic *ZmPYL*-overexpression lines were sterilized with 10% bleach and then kept at 4°C in the dark for 3 days. For the germination rate assay, sterilized seeds of wild-type and lines overexpressing *ZmPYLs* were sown on MS plates or MS plates supplemented with 1 μM ABA and grown under 16-h light: 8-h dark photoperiod at 22°C. Germination was first scored on the second day and counted continuously for 5 days. For the root growth assay, the seeds of wild-type and *ZmPYLs*-overexpression lines were sown perpendicularly on aaa MS agar medium and grown under 16-h light: 8-h dark photoperiod at 22°C for 5 days, then transferred to aaa MS agar medium containing 0 or 10 μM ABA. They were then grown vertically for 7 days, after which root lengths and leaf weights were measured. For the drought treatment, sterilized seeds of wild-type and *ZmPYL*-overexpression lines were sown on MS growth medium for 9 days, then transferred to small pots containing potting soil. *Arabidopsis* plants grew under normal watering conditions with 12 h light: 12 h dark photoperiod at 22°C for about 3 weeks. Watering was then halted. Plants were sampled for marker gene assays after 10 days when they began to exhibit lethal effects of dehydration. Samples were taken for proline and malondialdehyde (MDA) assays after 12 days. Watering was then resumed and the fraction surviving was determined.

### Identification and Analysis of *ZmPYLs*

All PYL protein sequences in Arabidopsis were obtained from the Ensemble Plants database footnotehttp://plants.ensembl.org/. To identify *ZmPYL* genes in maize, each Arabidopsis PYL sequence was analyzed by BLAST at maizeGDB hidetext^1^ against the B73 working gene set translations 5a.59 for RefGen_v2. After removing redundant results, all sequences were submitted to SMART^2^ to test whether the sequence had the Polyketide_cyc domain. Thirteen *ZmPYL* genes were retained for analysis in this study.

### Constructs

For *Arabidopsis* transformation, the 13 *ZmPYL* genes were individually cloned from cDNA of maize line B73. The cDNAs were sequenced after insertion into pJET1.2 vector. The *ZmPYL* cDNAs were then released from the pJET vectors by digestion with E*coR* I and X*ho* I, and inserted into the pSAT6 vector to produce pSAT6-ZmPYLs. The expression cassettes using 2X35S promoters to drive expression of the *ZmPYL* cDNAs were released from the pSAT6 vectors by digestion with P*I*-*Psp* I and inserted into the pRCS2-Bar-OCS binary vector (Dai et al., 2012).

### RNA Purification and Expression Analysis

Samples were collected after stress treatments. Total RNA was isolated using Trizol reagent (TransGen) from more than three seedlings for each treatment. RNA was treated with RNase-free DNase I (Thermo Scientific), and single-stranded cDNA was synthesized using recombinant M-MLV reverse transcriptase (Promega). The maize and Arabidopsis *Actin* genes were used as internal control to normalize the data.

### Proline and Malondialdehyde (MDA) Content

Arabidopsis seedlings that were dehydrated for 12 days were used for MDA and proline assays. To assay proline, 50 mg fresh weight (FW) of leaves were shredded into 10 ml centrifuge tubes using scissors. Five milliliters of 3% sulfosalicylic acid solution were added to each sample and then the mixture was placed in boiling water for 10–30 min to obtain the extract solution. After cooling to room temperature, 2 ml of supernatant were pipetted into a new 10 ml centrifuge tube and mixed with 2 ml acetic acid and 2 ml ninhydrin. The mixture was placed in a boiling water bath for 30 min. Four milliliters of methylbenzene were added to the extract after cooling to room temperature, followed by centrifugation for 1 min and stewing for 10 min. The upper red solution was transferred to a 1.5 ml centrifuge tube. After centrifugation at 12000 rpm for 15 min, the upper proline solution was pipetted into a cuvette to measure absorbance at 520 nm by UV-vis spectrophotometry with methylbenzene as blank.

To assay MDA content, 50 mg FW of leaves were shredded using scissors and placed in 5 ml 5% TCA. After centrifugation at 3000 rpm and 4°C for 10 min, 2 ml of the supernatants were transferred into new 10 ml centrifuge tubes then 2 ml 0.67% thiobarbituric acid (TBA) were added and the extract was placed in a boiling water bath for 30 min. After cooling to room temperature, 1.5 ml of the extracts were transferred into 1.5 ml tubes and centrifuged at 12000 rpm and room temperature. Absorbance of the supernatants were measured at 440, 532, and 600 nm by UV-vis spectrophotometry. The MDA content was calculated as nmol/g FW tissue. C/umol/L = 6.45 (A_532_-A_600_) -0.56A_450_

### Association Analyses

Association analyses for the *ZmPYL* genes were performed using a maize association mapping population that contains 368 inbred lines and corresponding drought tolerance phenotypic data obtained in a previous study. Among 525105 high-quality SNPs data with Minor Allele Frequency (MAF) ≥ 0.05, 112 SNPs were found in the regions of *ZmPYL3*, *ZmPYL8*, *ZmPYL9*, *ZmPYL10*, and *ZmPYL12* genes. The mixed linear model (MLM) was used to detect SNPs significantly associated with drought tolerance using the program TASSEL5.0 (Bradbury et al., 2007).

### Statistical Analyses

Statistical analyses were performed using Excel (Microsoft, United States). Figures were plotted by using Photoshop software (Adobe Systems, United States).

## Results

### Sequence Analyses of *ZmPYL* Genes

**FIGURE 1:**
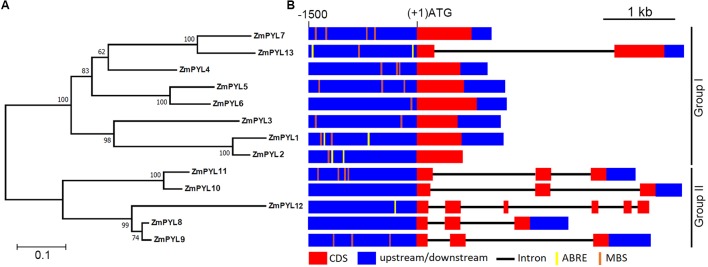
Evolutionary relationships of taxa. **(A)** Phylogenetic tree of ZmPYL proteins. The phylogenetic tree was generated based on the alignments of the full-length ZmPYL protein sequences. Bootstrap values from 1,000 replicates are indicated at each node. The scale bar represents branch lengths. **(B)** *ZmPYL* gene structures. The upstream regions (0∼–1500 bp) of these genes were used to search the public data base (PlantCARE). Most of the *ZmPYLs* except *ZmPYL8* and *ZmPYL10* contain one or more drought response elements (MBS or ABRE) in the upstream regions. MBS, MYB binding site; ABRE, ABA responsive element.

To determine how many genes encoding ZmPYL proteins are in the maize genome, each Arabidopsis PYL sequence was used as a BlastP query against the maize genome database (version B73 working gene set translations 5a.59 for RefGen_V2). Thirteen genes encoding ZmPYLs (*ZmPYL1*-*13*) were identified (Supplementary Table S1). These genes are located on most of the chromosomes with 1 or 2 genes per chromosome, except that no *ZmPYL* gene was detected on chromosome 7 (Supplementary Figure S1). A previous study reported 11 *ZmPYL* genes (Fan et al., 2016). The involvement of *ZmPYL3* in ABA signaling has been reported (Wang et al., 2014). To be consistent, the names of *ZmPYL1*-*11* in this study are same as those in the previous report (Fan et al., 2016). In our study, two more *ZmPYL* genes, GRMZM2G169695 (*ZmPYL13*, named in our study) and GRMZM2G405064 (*ZmPYL12*), were identified based on the conserved motifs and important amino acids of PYL family proteins (Li et al., 2013). Based on the protein sequences, a phylogenetic tree was generated using the NJ algorithm (**Figure 1A**). The ZmPYL proteins clustered into two subgroups, which is very similar to the Arabidopsis PYL proteins (**Figure 1A**; Li et al., 2013), suggesting the conservation of plant PYL proteins. Sequence analyses revealed that subgroup I genes had fewer introns than subgroup II *ZmPYL* genes (**Figure 1B**). In the promoter regions, a number of ABA responsive elements (ABREs) and MYB binding sites (MBS), which are involved in ABA and drought response (Seo et al., 2011; Singh and Laxmi, 2015), were detected in most *ZmPYL* genes except *ZmPYL8* and *ZmPYL10* (**Figure 1B**). The full-length coding sequences of the *ZmPYL* genes were cloned and confirmed by sequencing for further analyses.

### Expression Analyses of *ZmPYL* Genes

**FIGURE 2:**
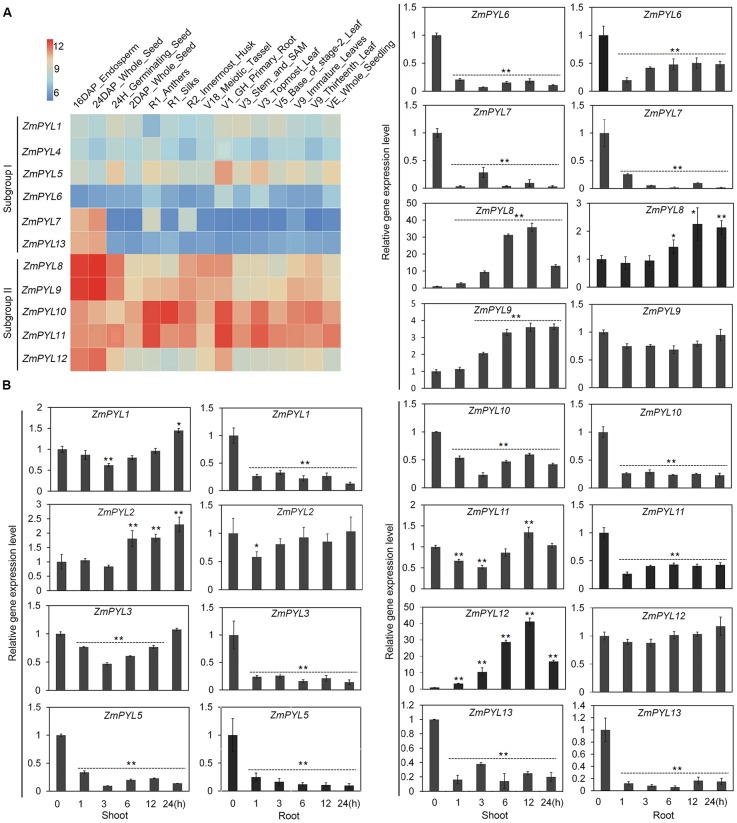
Expression Patterns of Maize *ZmPYL* Genes. **(A)** Gene expression heat map of 11 *ZmPYL* genes in 15 different tissues from various developmental stages. Two *ZmPYL* genes have no available data. The gene expression levels are shown in different colors indicated by the scale bar. **(B)** Expression patterns of *ZmPYL* genes in maize B73 leaves and roots under normal and drought treatments. *ZmActin5* gene was used as an internal control. Both leaf and root tissues were collected after treatment at time points 0, 1, 3, 6, 12, and 24 h. Data represent the mean ± SD of three biological replicates. Asterisks indicate the significance of *T*-test, ^∗^*p* < 0.05, ^∗∗^*p* < 0.01.

In order to determine the functions of *ZmPYL* genes in plant development, we studied their tissue-specific expression patterns. An expression heatmap of *ZmPYL* genes was created using the publically available transcription data for fifteen maize tissues (**Figure 2A**). According to the heatmap, the expression levels of *ZmPYL* genes were divided into two patterns. Subgroup II genes showed higher expression levels than those of subgroup I in most tissues (**Figure 2A**), indicating that subgroup II *ZmPYL* genes may have more important roles in regulating plant development. There are no expression data for *ZmPYL2* and *ZmPYL3* in the public database, therefore these genes were not included in the heatmap. Next we measured the expression levels of these genes in maize seedlings subjected to various levels of drought stress. *ZmPYL4* was not detectable in our experiments, may be due to the extremely low expression of this gene in the tissues tested. Based on the expression patterns, the genes can be roughly divided into three types (**Figure 2B**). Type I genes (*ZmPYL1*, *ZmPYL3*, and *ZmPYL11*) were slightly down-regulated initially but up-regulated at later stages of drought stress in shoots, while in roots they were down-regulated. Type II genes (*ZmPYL5*, *ZmPYL6*, *ZmPYL7*, and *ZmPYL10)*, were down-regulated in both shoots and roots after drought stress. Type III genes (*ZmPYL2*, *ZmPYL8*, *ZmPYL9*, and *ZmPYL12*) were up-regulated by drought in shoots and slightly up-regulated or unchanged in roots. These results suggested different roles of *ZmPYL* genes in regulation of drought responses. We next tested the responses of *ZmPYL* genes to other abiotic stresses, including ABA and salt stress. The results showed that ABA inhibited the expression of most *ZmPYL* genes in shoots and roots except for *ZmPYL7* and *ZmPYL8,* whose expression levels were enhanced in either roots (*ZmPYL8*) or both shoots and roots (*ZmPYL7*) (Supplementary Figure S2). After salt stress, the expression levels of *ZmPYL1*, *ZmPYL2*, *ZmPYL3*, *ZmPYL5*, *ZmPYL6, ZmPYL7*, *ZmPYL10*, and *ZmPYL13* were down-regulated, while the expression levels of *ZmPYL8*, *ZmPYL9*, *ZmPYL11*, and *ZmPYL12* were slightly up-regulated or not changed (Supplementary Figure S3). Taken together, these results suggested diverse roles of *ZmPYL* genes in regulation of maize abiotic stress responses.

### Roles of *ZmPYL* Genes in Regulation of ABA Responses

**FIGURE 3:**
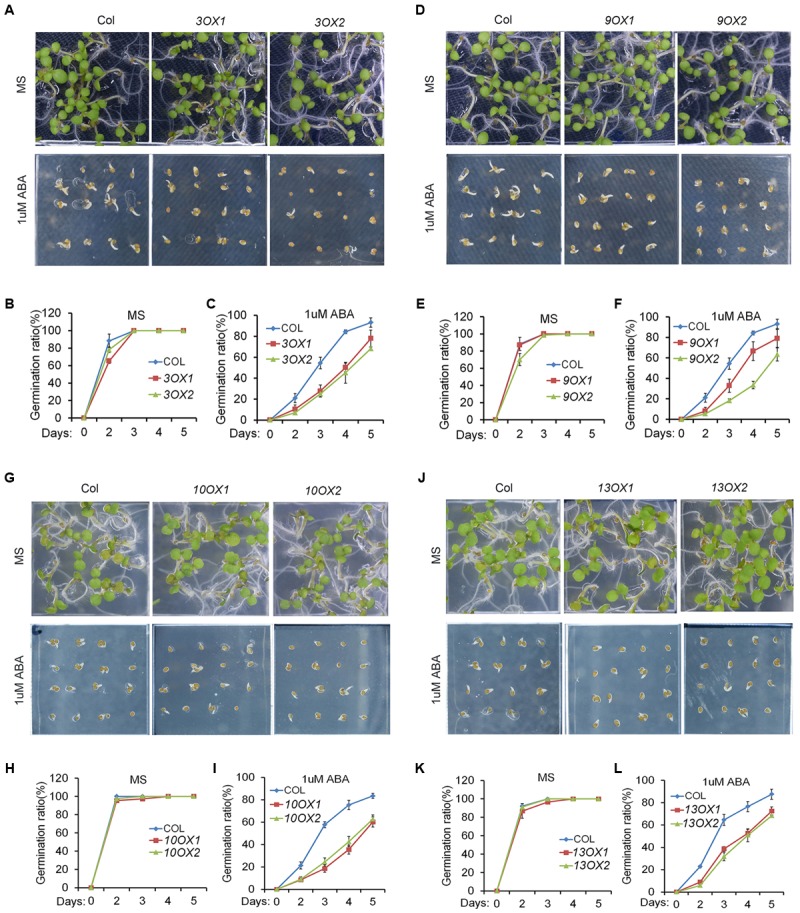
Overexpression of *ZmPYL3*, *ZmPYL9*, *ZmPYL10*, *ZmPYL13* in *Arabidopsis* increased the sensitivity of seed germination to abscisic acid (ABA). **(A–C)** Germination phenotypes **(A)** and statistical analyses **(B,C)** of Col and two transgenic seeds overexpressing *ZmPYL3* sown on MS medium or MS medium supplemented with 1 μM ABA. **(D–F)** Germination phenotypes **(D)** and statistical analyses **(E,F)** of Col and two transgenic seeds overexpressing *ZmPYL9* sown on MS medium or MS medium supplemented with 1 μM ABA. **(G–I)** Germination phenotypes **(G)** and statistical analyses **(H,I)** of Col and two transgenic seeds overexpressing *ZmPYL10* sown on MS medium or MS medium supplemented with 1 μM ABA. **(J–L)** Germination phenotypes **(J)** and statistical analyses **(K,L)** of Col and two transgenic seeds overexpressing *ZmPYL13* sown on MS medium or MS medium supplemented with 1 μM ABA. Data represent the mean ± SD of three replicates in **(B)**, **(C)**, **(E)**, **(F)**, **(H)**, **(I)**, **(K)**, and **(L)**.

**FIGURE 4:**
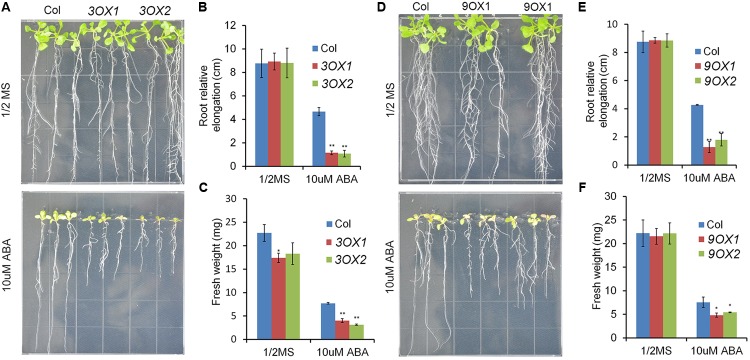
Overexpression of *ZmPYL3*, *ZmPYL9* in *Arabidopsis* increased the sensitivity of plant growth to ABA. **(A–C)** Root growth phenotypes **(A)**, statistical analyses of root elongation **(B)**, and fresh weigh **(C)** of Col and *ZmPYL3* overexpression transgenic lines grown on aaa MS medium or aaa MS medium containing ABA (10 μM). **(D–F)** Root growth phenotypes **(D)**, statistical analyses of root elongation **(E),** and fresh weigh **(F)** of Col and *ZmPYL9* overexpression transgenic lines grown on aaa MS medium or aaa MS medium containing ABA (10 μM). Five-day seedlings were transferred from aaa MS medium to aaa MS medium containing ABA (10 μM), then grown vertically for 7 days before phenotype. Data represent the mean ± SD of three replicates for **(B)**, **(C)**, **(E)**, and **(F)**. Asterisks indicate the significance of *T*-test, ^∗^*p* < 0.05, ^∗∗^*p* < 0.01.

In order to decipher the biological functions of *ZmPYL* genes, we generated transgenic Arabidopsis plants overexpressing the *ZmPYL* genes. A total of 12 *ZmPYL* genes were introduced into and overexpressed in Arabidopsis plants except *ZmPYL4*. Our failure to clone *ZmPYL4* may be due to its extremely low expression in most of maize tissues. In order to determine how these *ZmPYL* genes function in regulating ABA responses, we germinated *ZmPYL* transgenic seeds on MS plates with or without ABA, and then measured the germination rates at various time points. To our surprise, we only observed that transgenic seeds overexpressing *ZmPYL3*, *ZmPYL9*, *ZmPYL10,* and *ZmPYL13* responded to ABA treatment (**Figure 3** and Supplementary Figure S4). These transgenic seeds were hypersensitive to ABA, suggesting negative roles of *ZmPYL3*, *ZmPYL9*, *ZmPYL10,* and *ZmPYL13* in regulation of ABA-mediated seed germination. It is well known that ABA inhibits plant growth (Luo et al., 2014). To determine how *ZmPYL3*, *ZmPYL9*, *ZmPYL10,* and *ZmPYL13* regulate plant growth in response to ABA, we grew the control and transgenic seedlings on MS plates with or without ABA, and then measured the root lengths and FW. The results showed that the transgenic seedlings overexpressing *ZmPYL3* or *ZmPYL9* had more severely inhibited plant growth than the controls, suggesting that they were more sensitive to ABA than the controls (**Figure 4**). These results indicated that the activities of *ZmPYL3* and *ZmPYL9* were critical for both seed germination and post-germination growth. Intriguingly, we did not observe any phenotypic changes in *ZmPYL13* and *ZmPYL10* transgenic plants as compared to controls after ABA treatment (Data not shown), suggesting a specific role of *ZmPYL10* and *ZmPYL13* in regulating Arabidopsis seed germination rather than seedling growth.

### Roles of *ZmPYL* Genes in Regulation of Drought Responses

**FIGURE 5:**
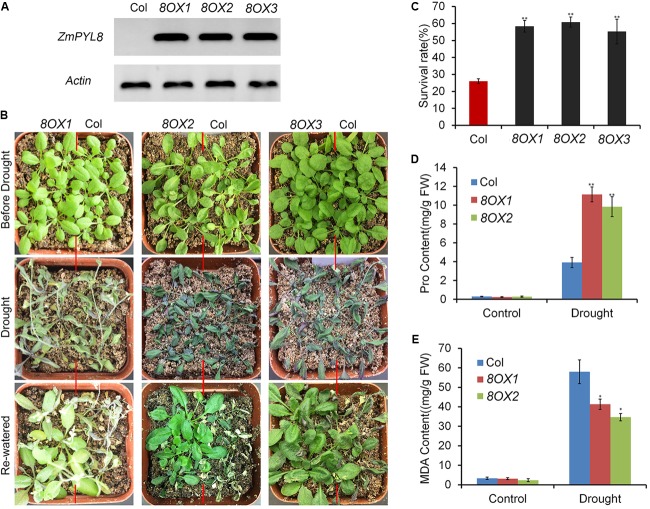
Overexpression of *ZmPYL8* in *Arabidopsis* enhanced drought tolerance. **(A)** RT-PCR shows overexpression of *ZmPYL8* (8OX) in three independent transgenic lines. *Arabidopsis actin7* gene was used as control. **(B)** Drought tolerance assay of three transgenic lines overexpressing *ZmPYL8*. Three-week old plants were drought-stressed for 2 weeks and then rewatered for 1 day. **(C)** Survival rates of Col and *ZmPYL8* transgenic plants after drought stress. Data represent the mean ± SD of three replicates. **(D)** Proline contents of watered and drought-stressed Col and *ZmPYL8* transgenic plants. **(E)** Malondialdehyde (MDA) contents of watered and drought-stressed Col and *ZmPYL8* transgenic plants. Data represent the mean ± SD of three biological replicates. Asterisks indicate the significance of *T*-test, ^∗^*p* < 0.05, ^∗∗^*p* < 0.01.

Next, we performed experiments to test how *ZmPYL* genes regulate plant drought responses. All the transgenic lines overexpressing *ZmPYL* genes were subjected to severe drought stress. After stress, the plants were re-watered and survival rates of the transgenic lines were determined. We observed that most plants overexpressing *ZmPYL8* recovered upon rewatering after severe drought stress, whereas most control plants died (**Figures 5A,B**). The survival rates of plants overexpressing *ZmPYL8* were twofold higher than those of controls (**Figure 5C**). Further analyses showed that more than twofold higher of proline (**Figure 5D**), and 30% less of MDA accumulated in plants overexpressing *ZmPYL8* after drought stress as compared to controls (**Figure 5E**). These results suggested that *ZmPYL8* plays a positive role in regulation of plant drought tolerance. We observed similar phenotypic changes in plants overexpressing *ZmPYL9* and *ZmPYL12*. After drought stress, the transgenic plants overexpressing *ZmPYL9* and *ZmPYL12* had higher survival rates than the controls (Supplementary Figure S5), suggesting that *ZmPYL9* and *ZmPYL12* also have positive roles in plant drought tolerance.

**FIGURE 6:**
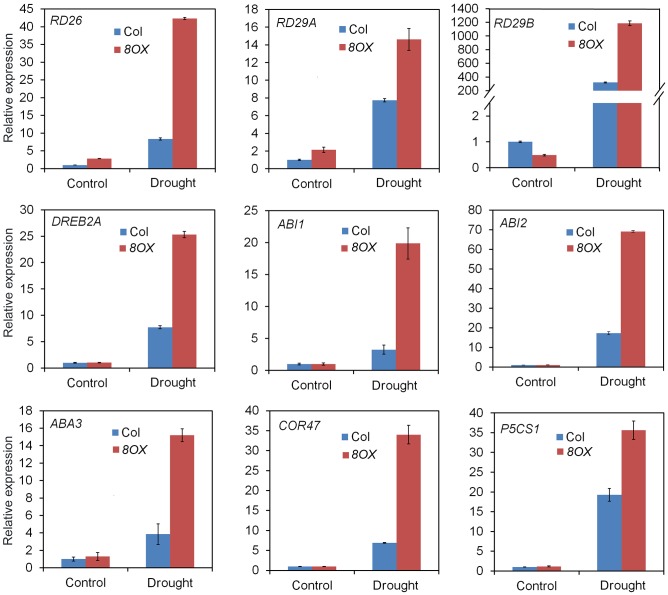
Overexpression of *ZmPYL8* raised the expression of drought responsible marker genes. The expression of all marker genes under normal and drought conditions was examined by qRT-PCR. Three-week old plants were either watered or drought-stressed for 10 days.

When compared to control plants, we did not observe any phenotypic changes in transgenic plants overexpressing other *ZmPYL* genes in our drought stress experiments (Data not shown), suggesting these genes do not play major roles in regulating drought resistance of the transgenic plants. *ZmPYL8*, *ZmPYL9*, and *ZmPYL12* clustered together in the phylogenetic tree (**Figure 1A**), suggesting conserved roles of these genes at least in drought resistance. We therefore chose plants overexpressing *ZmPYL8* as representatives to measure the expression of drought-responsive marker genes. The results showed that the expression of all of these marker genes was enhanced in transgenic plants after drought stress (**Figure 6**). The increased expression of *ABA3*, *COR47*, *RD26*, *RD29A*, *RD29B*, *ABI1*, *ABI2*, *DREB2A*, which are known to respond to ABA, suggested enhanced ABA signaling in transgenic plants. P5CS1 is an enzyme responsible for proline biosynthesis (Kesari et al., 2012). The enhanced expression of *P5CS1* is consistent with the elevated accumulation of proline in *ZmPYL8* transgenic plants (**Figure 5D**). Taken together, these results suggested overexpression of *ZmPYL8* promoted ABA signaling, which, in turn, enhanced drought resistance of the transgenic plants.

### Identification of Natural Variation in *ZmPYL* Genes Associated With Drought Resistance

**FIGURE 7:**
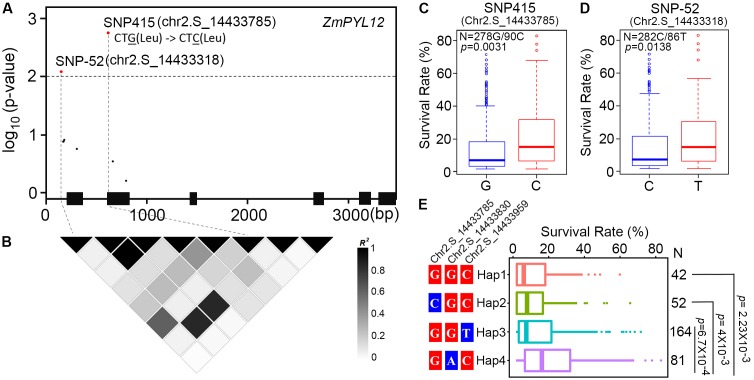
Association analysis of genetic variations in *ZmPYL12* with maize drought tolerance. **(A)** Associations of *ZmPYL12* SNPs with drought tolerance. The gene structure is shown at the bottom. The significant associations are shown in red (*p* < 0.01). Exons and introns are shown as filled boxes and dark lines, respectively. SNP415 has G/C natural variations, but does not change the coding information of Leu. **(B)** LD map for *ZmPYL12* SNPs. Color in each block represents the R-squared value between two SNPs. **(C)** Association of two alleles in SNP415 with drought tolerance. **(D)** Association of two alleles in SNP-52 with drought tolerance. **(E)** Association of hyplotypes in *ZmPYL12* with drought tolerance.

Previous studies have identified one million SNPs in a maize panel consisting of 368 accessions (Fu et al., 2013). Using survival rates after severe drought stress as indices, the drought-resistance phenotypes of these maize accessions were reported in several studies (Liu et al., 2013; Wang et al., 2016). To determine whether natural variation in *ZmPYL* genes was associated with maize drought resistance, we performed candidate gene association analyses with the MLM on *ZmPYL3*, *ZmPYL8*, *ZmPYL9*, *ZmPYL10*, *ZmPYL12*, which we identified as playing roles in regulation of ABA responses and/or drought resistance (**Figures 3**–**5** and Supplementary Figure S5). By using the genotypic and phenotypic data of the 368 accessions, we observed from association analyses that two genes, *ZmPYL8* and *ZmPYL12*, had natural variants that were significantly associated with drought resistance (*p* ≤ 0.01, **Table 1**). The most significant *p*-value (lead *p*-value) was 0.00277 for *ZmPYL8* and 0.0018 for *ZmPYL12* (**Table 1**). Further analyses showed that the lead SNP (SNP1543, with lead *p*-value) in *ZmPYL8* was located in the third exon of this gene, and showed strong LD with another significant SNP (SNP1640) located downstream of the lead SNP (Supplementary Figure S6). The lead SNP of *ZmPYL12* (SNP415) was located in the second exon and showed strong LD with another significant SNP (SNP-52) located upstream of the lead SNP (**Figures 7A,B**). Further analyses identified the drought-resistant alleles of SNP415 and SNP-52 in *ZmPYL12* (**Figures 7C,D**). In the association populations, there were four major haplotypes (Hap), among which Hap4 was most drought-resistant (**Figure 7E**). In *ZmPYL8*, we did not detect drought-resistant alleles from the associated SNPs (data not shown).

**Table 1:** Natural variations in *ZmPYL* genes associated with drought resistance in 368 maize inbred lines.

Gene ID	Name	Function	Polymorphic number^∗^	MLM (*p* ≤ 0.01)	Lead *p*-value
GRMZM2G154987	*ZmPYL3*	ABA	22	0	0.2909
GRMZM2G165567	*ZmPYL8*	Drought	40	2	0.00277
GRMZM2G133631	*ZmPYL9*	ABA, drought	26	0	0.04244
GRMZM2G063882	*ZmPYL10*	ABA	16	0	0.0436
GRMZM2G405064	*ZmPYL12*	Drought	8	2	0.0018

^∗^*MAF (Minor Allele Frequency) ≥ 0.05; ABA, abscisic acid; MLM, mixed linear model*.

## Discussion

Abscisic acid is well-known to be an important phytohormone regulating plant stress responses. A recent breakthrough in our understanding of ABA signaling came when two different groups identified the ABA receptors, the RCAR/PYL family proteins (Ma et al., 2009; Park et al., 2009). Subsequently, the functions of PYL receptors in regulation of ABA and stress responses have been widely studied (Klingler et al., 2010; Antoni et al., 2013; Liang et al., 2017; Pri-Tal et al., 2017). Maize is an important food and industrial crop worldwide and every year suffers yield losses due to drought. How ZmPYL ABA receptors are involved in maize drought resistance remains largely unknown. In this study, we comprehensively investigated the expression of *ZmPYL* genes in response to ABA and drought stresses, the roles of *ZmPYLs* in regulating ABA and drought responses and natural variants of *ZmPYL* genes associated with maize drought resistance.

Thirteen *ZmPYLs* genes were identified in this study. Analyses of the promoter sequences of these genes identified multiple *cis* elements, including ABRE and MBS, which have been reported to be involved in responses to ABA and drought stress (Seo et al., 2011; Singh and Laxmi, 2015). The changes in *ZmPYL* gene expression after ABA and drought stresses indicated the roles of these *cis* elements in regulation of gene expression. Intriguingly, most *ZmPYL* genes were down-regulated after drought treatment, whereas *ZmPYL8*, *ZmPYL9*, *ZmPYL2*, and *ZmPYL12* were up-regulated. After ABA treatment, most *ZmPYL* genes were down-regulated, while *ZmPYL7* gene was up-regulated. Upon salt treatment, about 50% *ZmPYL* genes were down-regulated, while the rest were slightly affected or not changed. The different response patterns of *ZmPYL* genes after drought, ABA and salt treatments suggested diverse roles of *ZmPYLs* in regulation of plant abiotic stress responses. A previous study reported the expression of 11 *ZmPYLs* in response to ABA and PEG treatments (Fan et al., 2016). Both previous and this studies revealed very similar expression patterns of most *ZmPYL* genes in response to ABA, except *ZmPYL1*-*3* (Supplementary Figure S2; Fan et al., 2016). The various expression patterns of few *ZmPYLs* may be caused by different growth stages or environments for the experiments. Interestingly, *ZmPYLs* showed very different expression patterns in response to PEG or drought treatments. For instance, the expression of half of *ZmPYLs* was down-regulated under drought in this study, but the expression of most of *ZmPYLs* was up-regulated in response to PEG (**Figure 2**; Fan et al., 2016). These results suggested different effects of PEG and drought on *ZmPYL* expression.

To investigate the biological functions of *ZmPYLs*, we cloned all of the *ZmPYL* genes and overexpressed them in Arabidopsis plants, except *ZmPYL4* due to failure of detecting its expression in maize leaves. We observed that overexpression of four genes, *ZmPYL3*, *ZmPYL9*, *ZmPYL10*, and *ZmPYL13*, resulted in hypersensitivity to ABA treatment, suggesting negative roles of these genes in ABA responses in transgenic plants. This is very similar to what was observed in Arabidopsis, where *PYL* genes also played negative roles in ABA responses (Ma et al., 2009; Park et al., 2009), suggesting that the negative roles of these *PYL* genes in ABA responses are conserved across various plant species. There are 13 *ZmPYL* genes in maize, but surprisingly only four of them had roles in regulating ABA responses when ectopically expressed in Arabidopsis. One possibility is that the other *ZmPYL* genes have other roles rather than mediating ABA signaling. Considering that the expression levels of *ZmPYL1*, *ZmPYL4*, *ZmPYL5*, and *ZmPYL6* were also dramatically inhibited by ABA in maize but played no roles in regulation of ABA responses when ectopically expressed in Arabidopsis, another possible reason is that these *ZmPYL* genes may not function in Arabidopsis. Transgenic studies of these *ZmPYL* genes in maize may provide more precise evaluation of their roles in plant development and ABA responses.

In drought stress experiments, we observed that overexpressing *ZmPYL8*, *ZmPYL9*, and *ZmPYL12* in Arabidopsis, resulted in resistance of the transgenic plants to drought treatment, suggesting positive roles of these genes in drought responses. *ZmPYL8*, *ZmPYL9*, and *ZmPYL12* were clustered together in the phylogenetic tree (**Figure 1**). After drought treatment, *ZmPYL8*, *ZmPYL9*, and *ZmPYL12* showed very similar expression patterns in both shoots and roots (**Figure 2**). These observations indicated conserved roles of these genes in regulation of plant drought resistance. But the fact that only *ZmPYL9* was involved in regulation of both drought and ABA responses indicated functional diversity of these genes. A previous study reported the roles of Arabidopsis *PYLs* in plant drought stress responses by ectopically expressing these genes with various promoters, including the stress-inducible *RD29A* promoter, the *GC1* and *ROP11* promoters (guard cell-specific) and the *RBCS* promoter (green tissue-specific), as well as the constitutive 35S promoter (Zhao et al., 2016). Based on their experiments, different promoters resulted in different levels of drought resistance when these promoters were used to drive the expression of various *PYL* genes (Zhao et al., 2016), indicating that the roles of plant *PYLs* in drought resistance depends on their temporal and spatial patterns of expression. In future, it will be worthwhile to use maize stress-inducible or tissue-specific promoters to study the functions of *ZmPYL* genes in regulation of drought resistance.

Developing new molecular markers is one of the most important topics in plant breeding. Thanks to the advances in high-throughput technologies, millions of SNP markers distributed throughout the whole genome at high density have been developed for various crops, including maize, rice, and other crops (Huang et al., 2011; Jiao et al., 2012; Fu et al., 2013; Zhou et al., 2015; Yano et al., 2016). Candidate gene association analyses, which are based on genetic variants and plant phenotypes, are widely used in dissecting the natural variation involved in plant drought resistance (Liu et al., 2013; Xiang et al., 2017). By using this association method, we detected natural variants of both *ZmPYL8* and *ZmPYL12* from a maize panel consisting of 368 natural accessions. Considering that these associations were most significant as compared to those detected in other *ZmPYL* genes which regulated ABA responses, and that *ZmPYL8* and *ZmPYL12* were proved to play positive roles in plant drought resistance, these associations are not false positive but real associations. Further analyses failed to detect resistant alleles in *ZmPYL8*, perhaps because they are not causal alleles of drought resistance. However, we detected resistant alleles in *ZmPYL12* (**Figure 7**), indicating that these alleles are causal alleles or have strong LD with the causal alleles.

Together, our results revealed the fundamental roles *ZmPYLs* in mediating ABA signaling and drought resistance in maize. Breeding of drought-resistant maize cultivars is an important goal for maize breeders. The drought-resistant *ZmPYLs* may be used as genetic resources in drought-tolerant breeding via transgenic approaches. Moreover, the resistant alleles or haplotypes detected in *ZmPYL12* have the potential to be used as molecular markers in genetic improvement of maize drought resistance.

## Author Contributions

MD and ZH designed the experiments. ZH and JZ conducted the experiments and analyzed the data. XS helped in the association and bioinformatic analyses. BW helped in the transgenic experiments. WT carefully edited the manuscript and analyzed the data. MD, ZH, and JZ wrote the manuscript. All authors revised the manuscript.

## Conflict of Interest Statement

The authors declare that the research was conducted in the absence of any commercial or financial relationships that could be construed as a potential conflict of interest.

## Footnotes

1 http://www.maizegdb.org/

2 http://smart.embl-heidelberg.de/
