# Supplementary material for: The Maize ABA Receptors ZmPYL8, 9, and 12 Facilitate Plant Drought Resistance
Source: Front Plant Sci. 2018 Apr 4;9:422. doi: 10.3389/fpls.2018.00422 (PMC5893742; doi:10.3389/fpls.2018.00422)
Supplement: Supplementary file 2 [file Presentation_1.PPTX]

## Slide 1
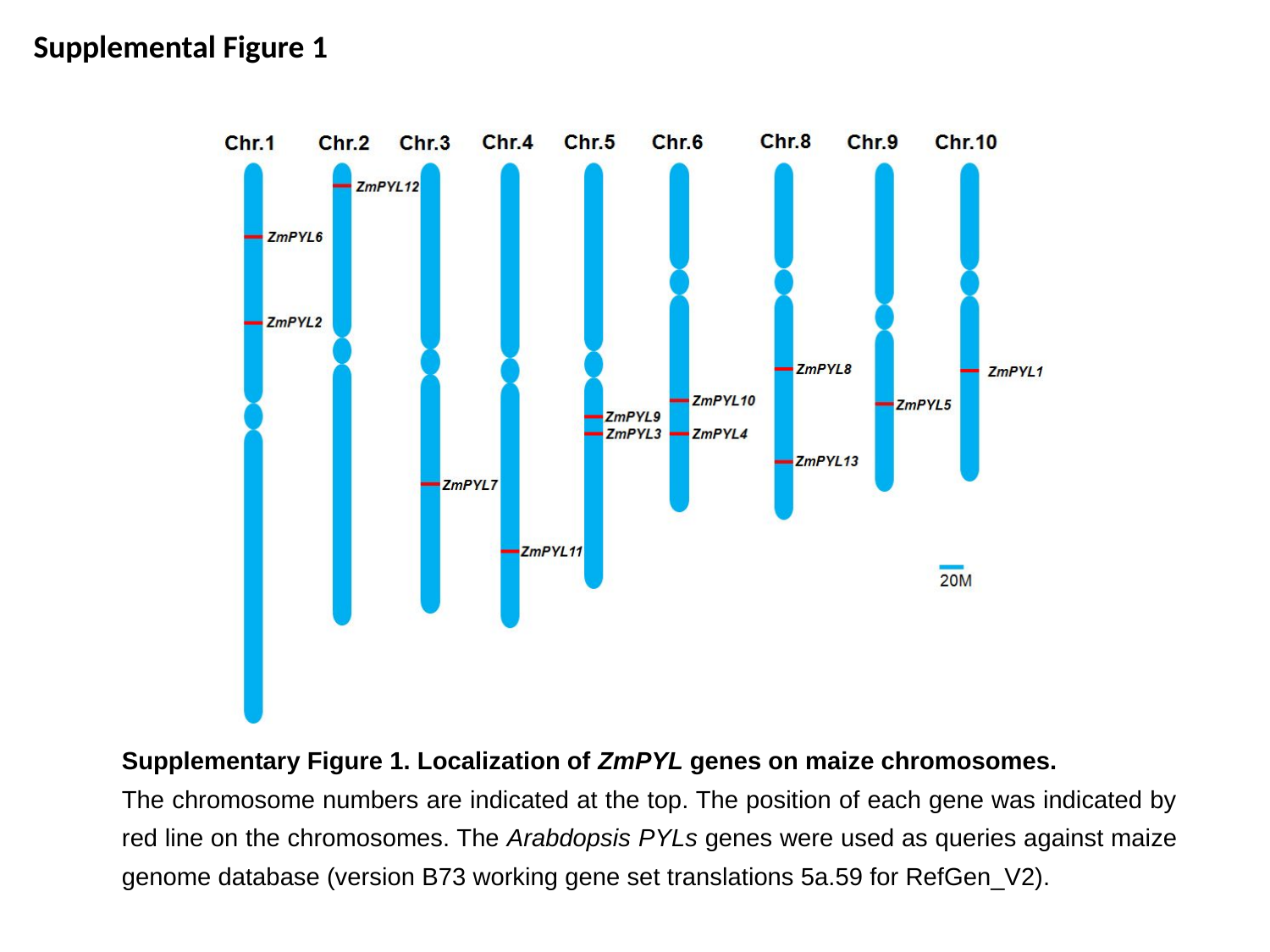

Supplemental Figure 1
Supplementary Figure 1. Localization of ZmPYL genes on maize chromosomes.
The chromosome numbers are indicated at the top. The position of each gene was indicated by red line on the chromosomes. The Arabdopsis PYLs genes were used as queries against maize genome database (version B73 working gene set translations 5a.59 for RefGen_V2).

## Slide 2
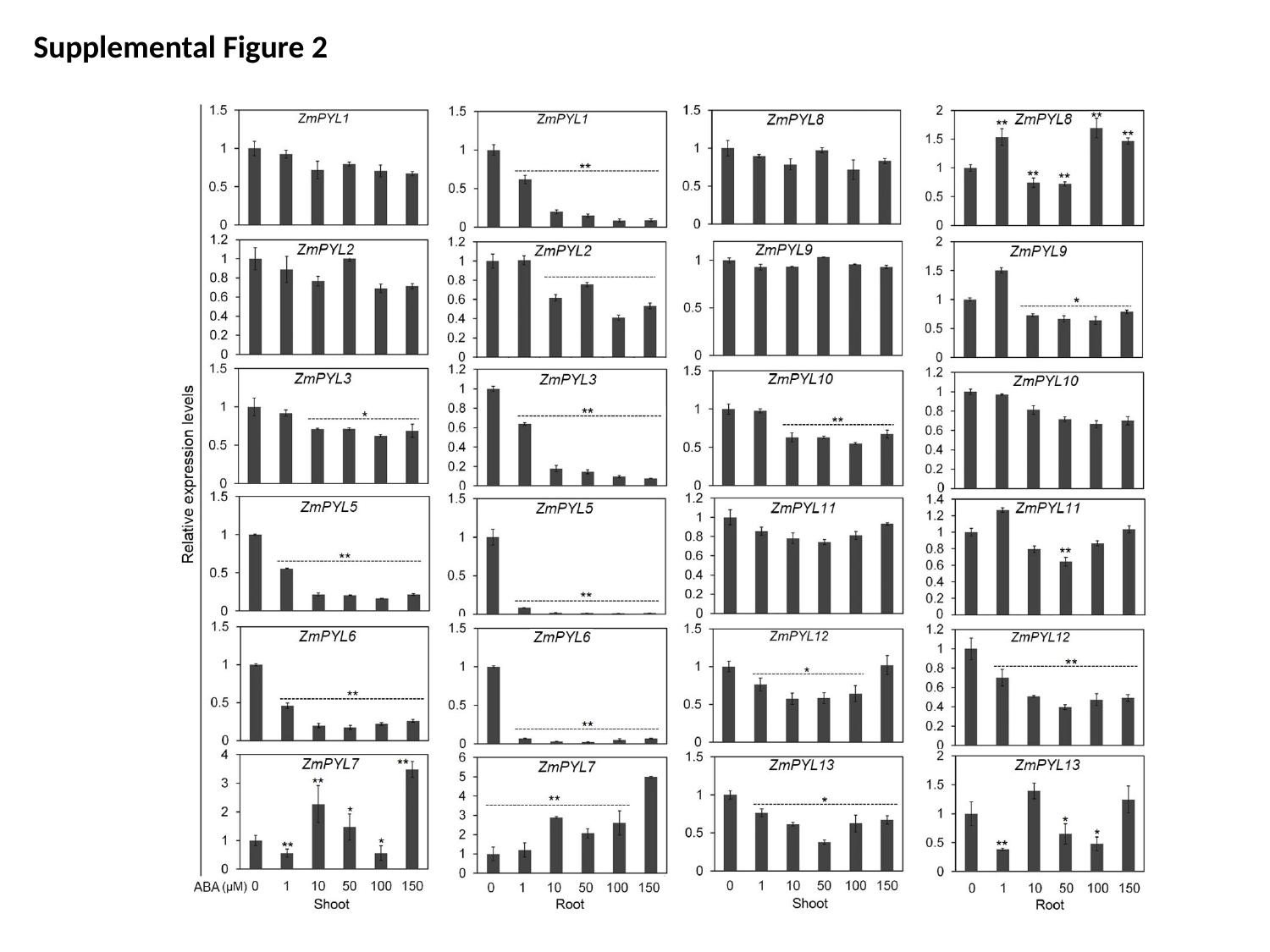

Supplemental Figure 2

## Slide 3
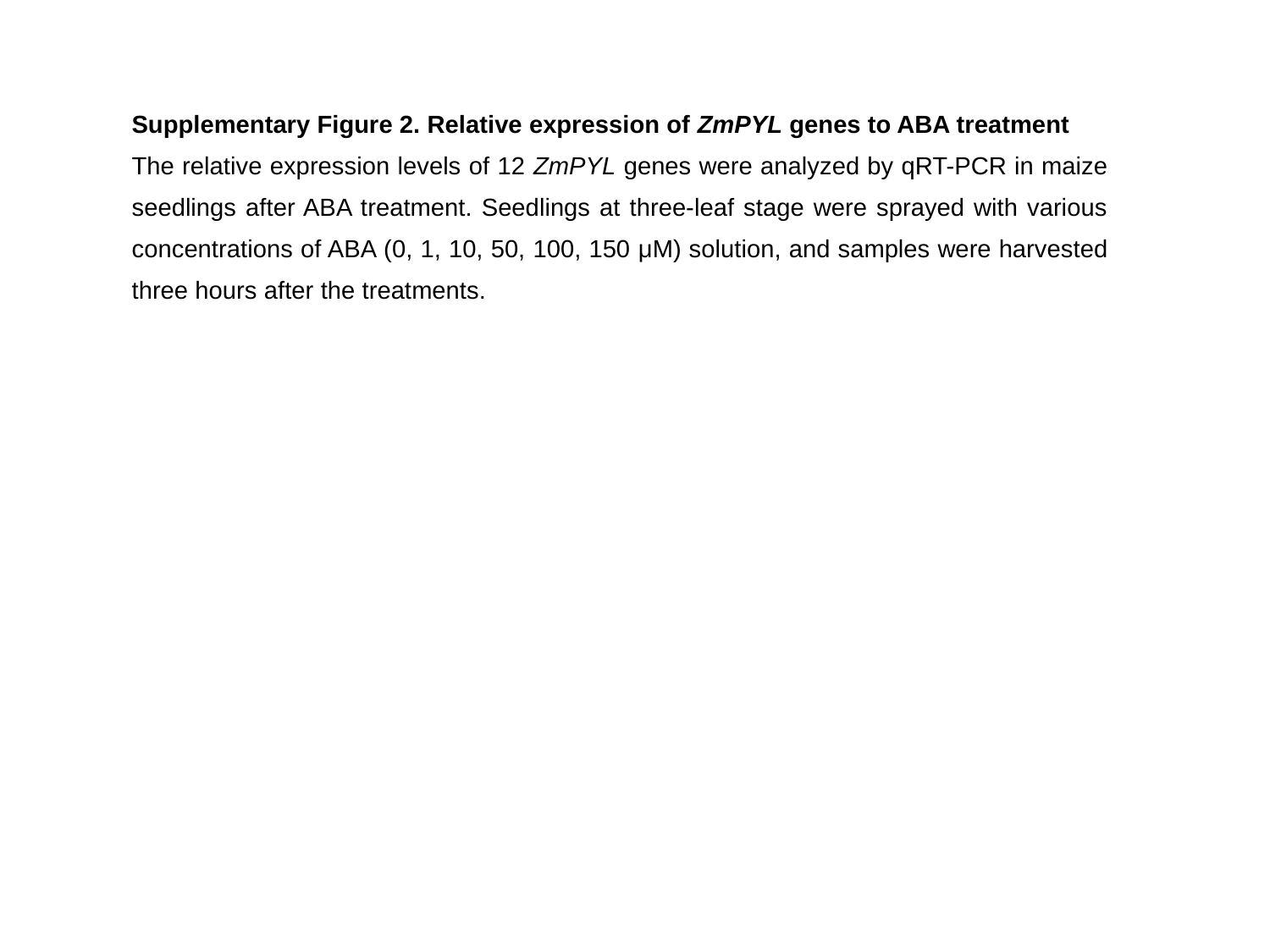

Supplementary Figure 2. Relative expression of ZmPYL genes to ABA treatment
The relative expression levels of 12 ZmPYL genes were analyzed by qRT-PCR in maize seedlings after ABA treatment. Seedlings at three-leaf stage were sprayed with various concentrations of ABA (0, 1, 10, 50, 100, 150 μM) solution, and samples were harvested three hours after the treatments.

## Slide 4
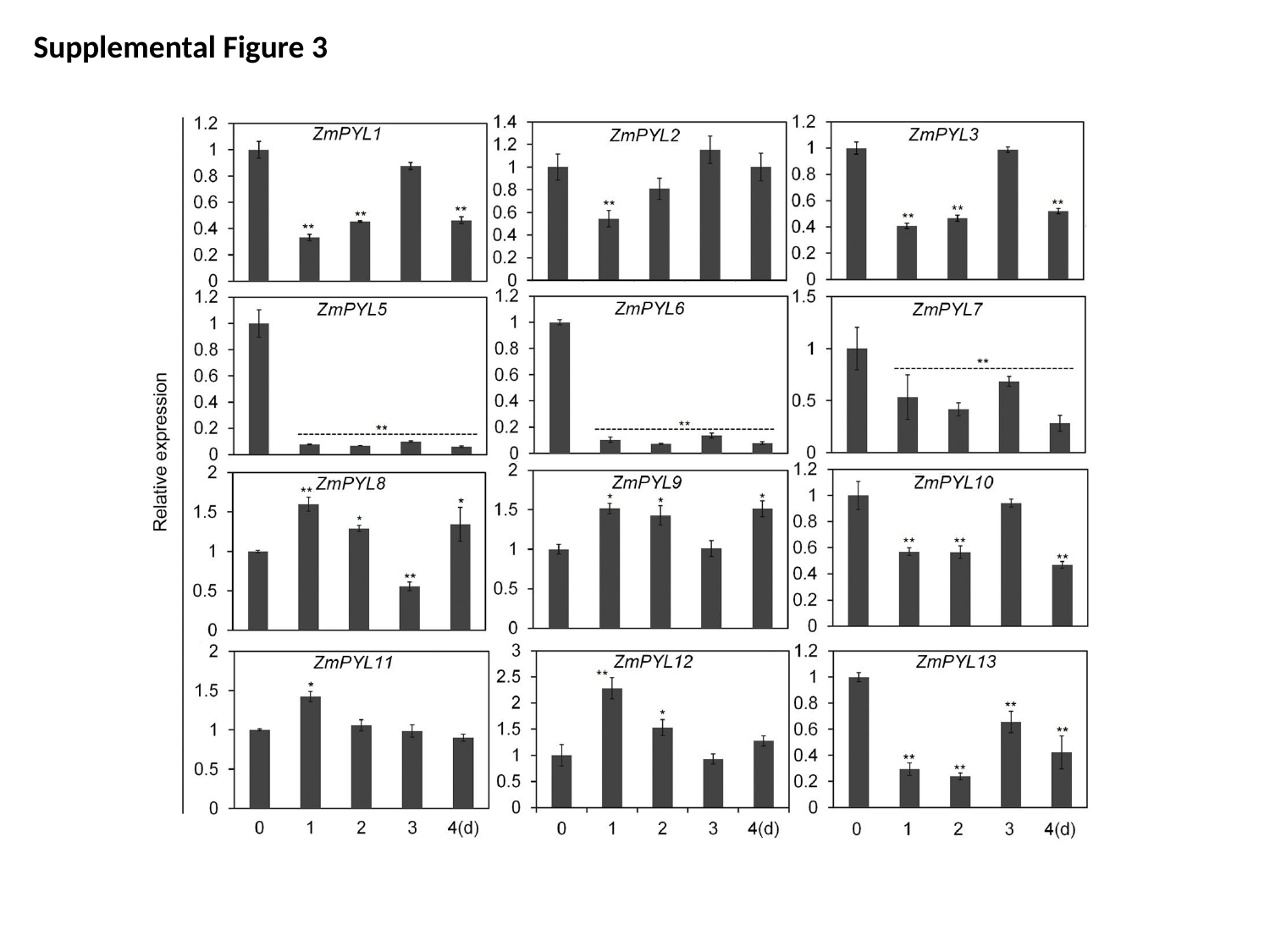

Supplemental Figure 3

## Slide 5
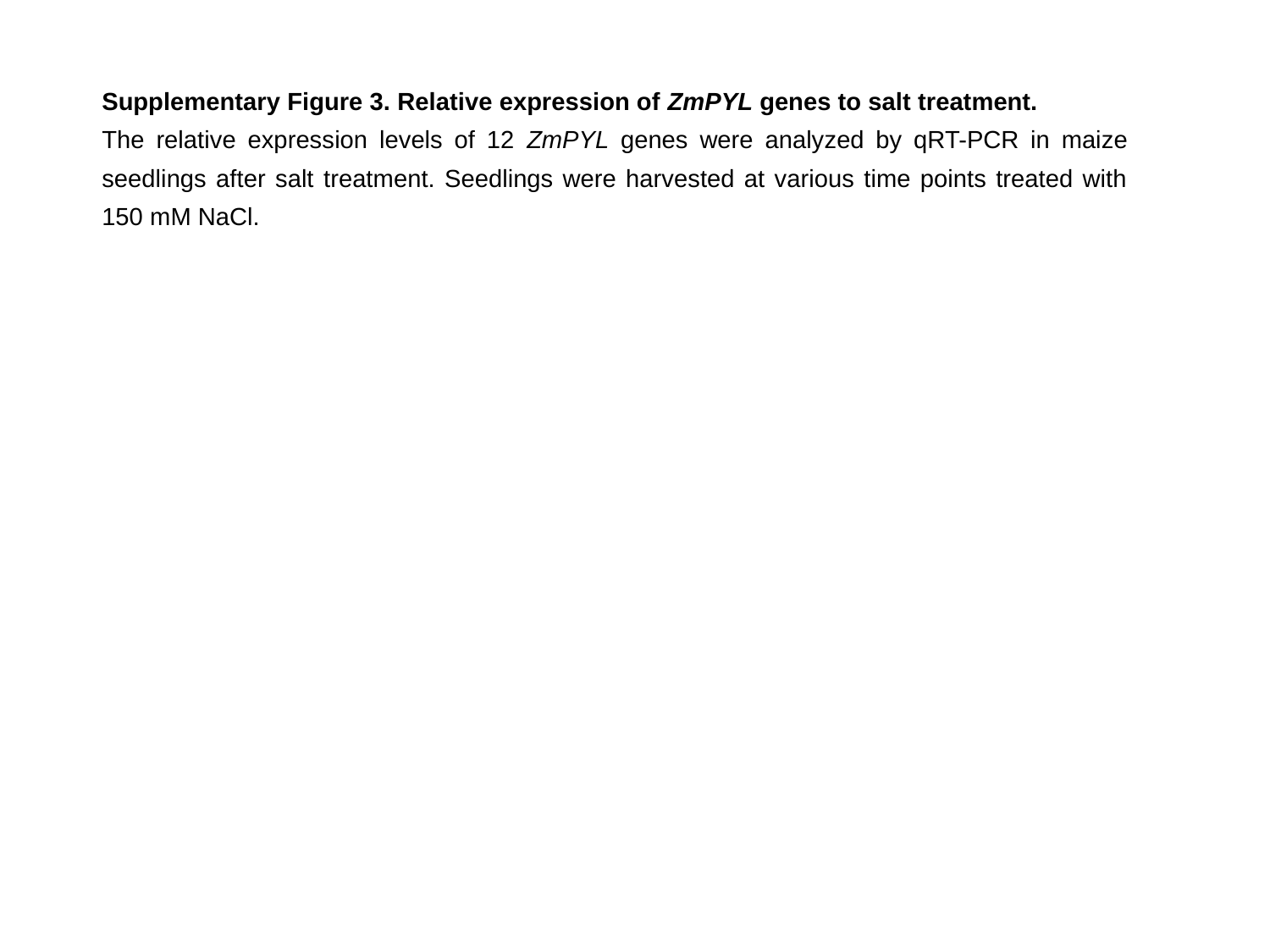

Supplementary Figure 3. Relative expression of ZmPYL genes to salt treatment.
The relative expression levels of 12 ZmPYL genes were analyzed by qRT-PCR in maize seedlings after salt treatment. Seedlings were harvested at various time points treated with 150 mM NaCl.

## Slide 6
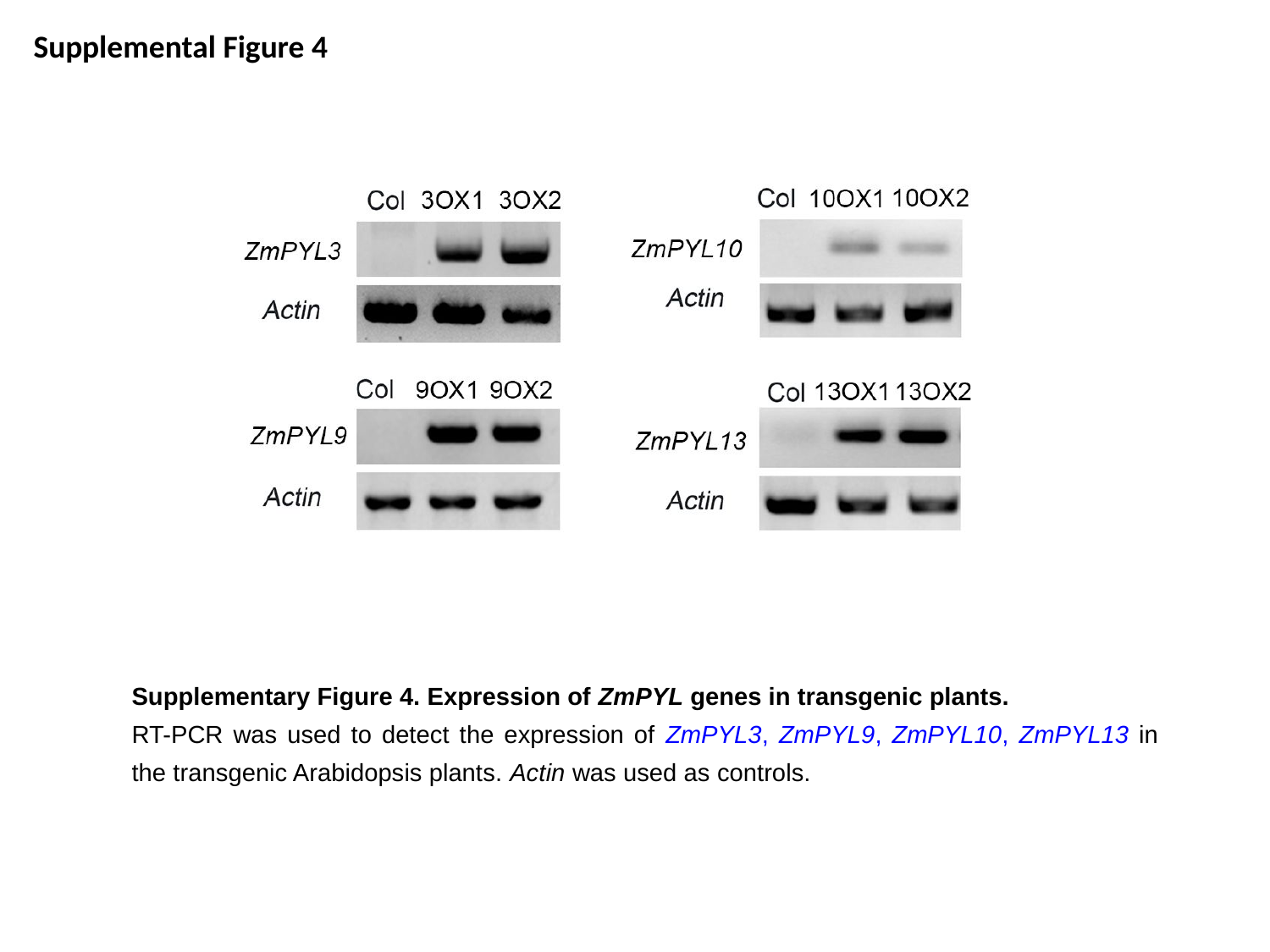

Supplemental Figure 4
Supplementary Figure 4. Expression of ZmPYL genes in transgenic plants.
RT-PCR was used to detect the expression of ZmPYL3, ZmPYL9, ZmPYL10, ZmPYL13 in the transgenic Arabidopsis plants. Actin was used as controls.

## Slide 7
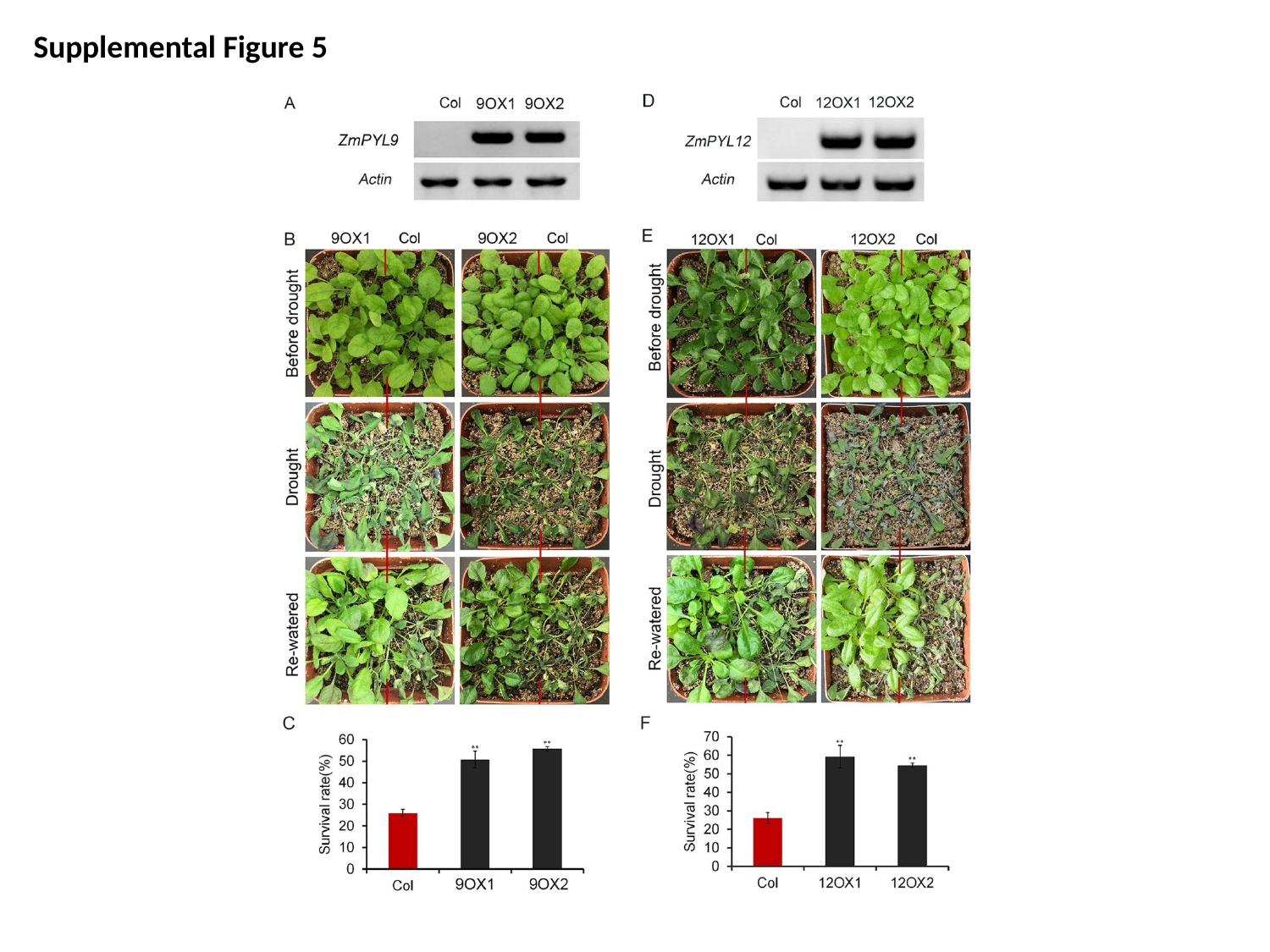

Supplemental Figure 5

## Slide 8
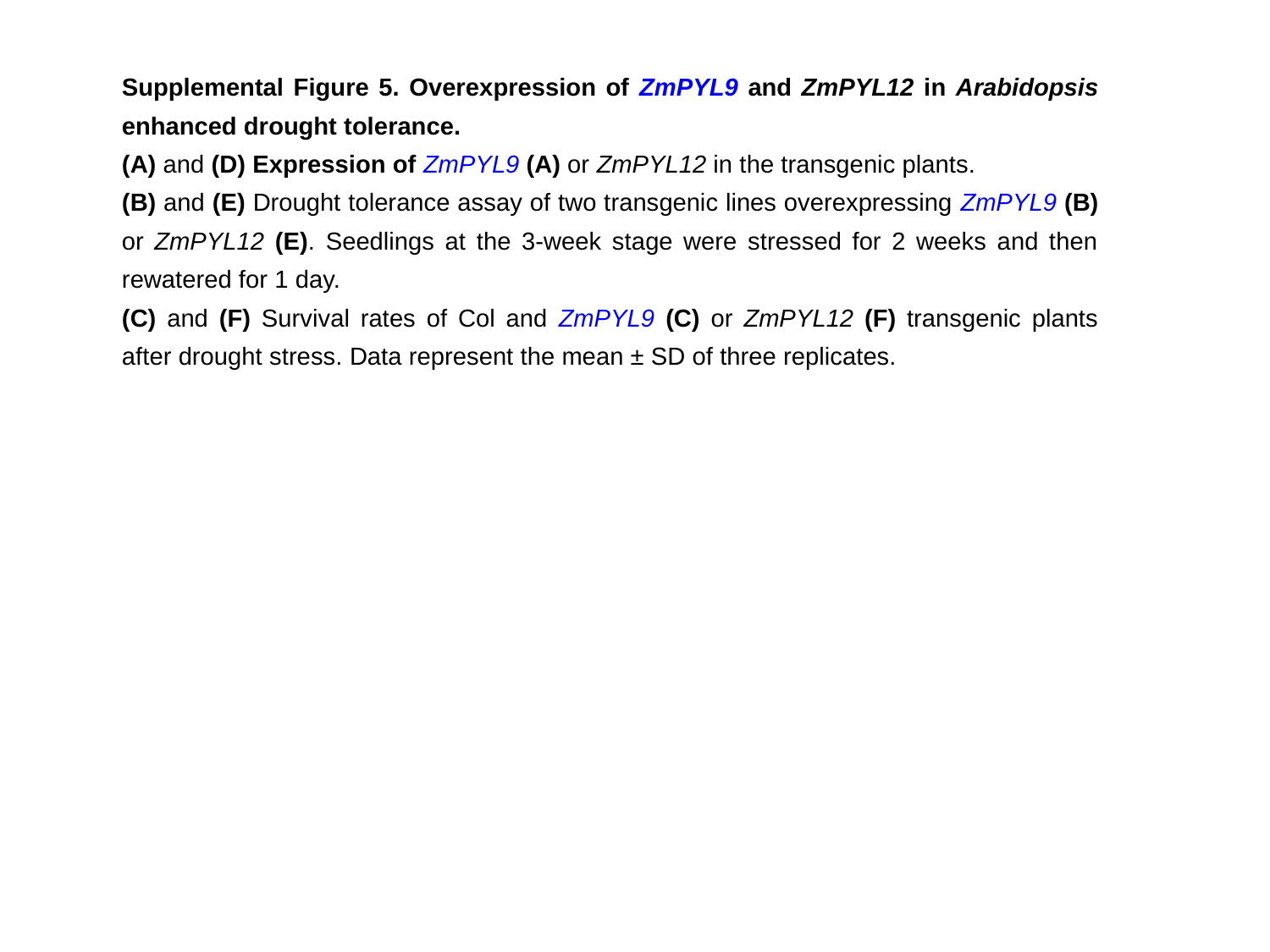

Supplemental Figure 5. Overexpression of ZmPYL9 and ZmPYL12 in Arabidopsis enhanced drought tolerance.
(A) and (D) Expression of ZmPYL9 (A) or ZmPYL12 in the transgenic plants.
(B) and (E) Drought tolerance assay of two transgenic lines overexpressing ZmPYL9 (B) or ZmPYL12 (E). Seedlings at the 3-week stage were stressed for 2 weeks and then rewatered for 1 day.
(C) and (F) Survival rates of Col and ZmPYL9 (C) or ZmPYL12 (F) transgenic plants after drought stress. Data represent the mean ± SD of three replicates.

## Slide 9
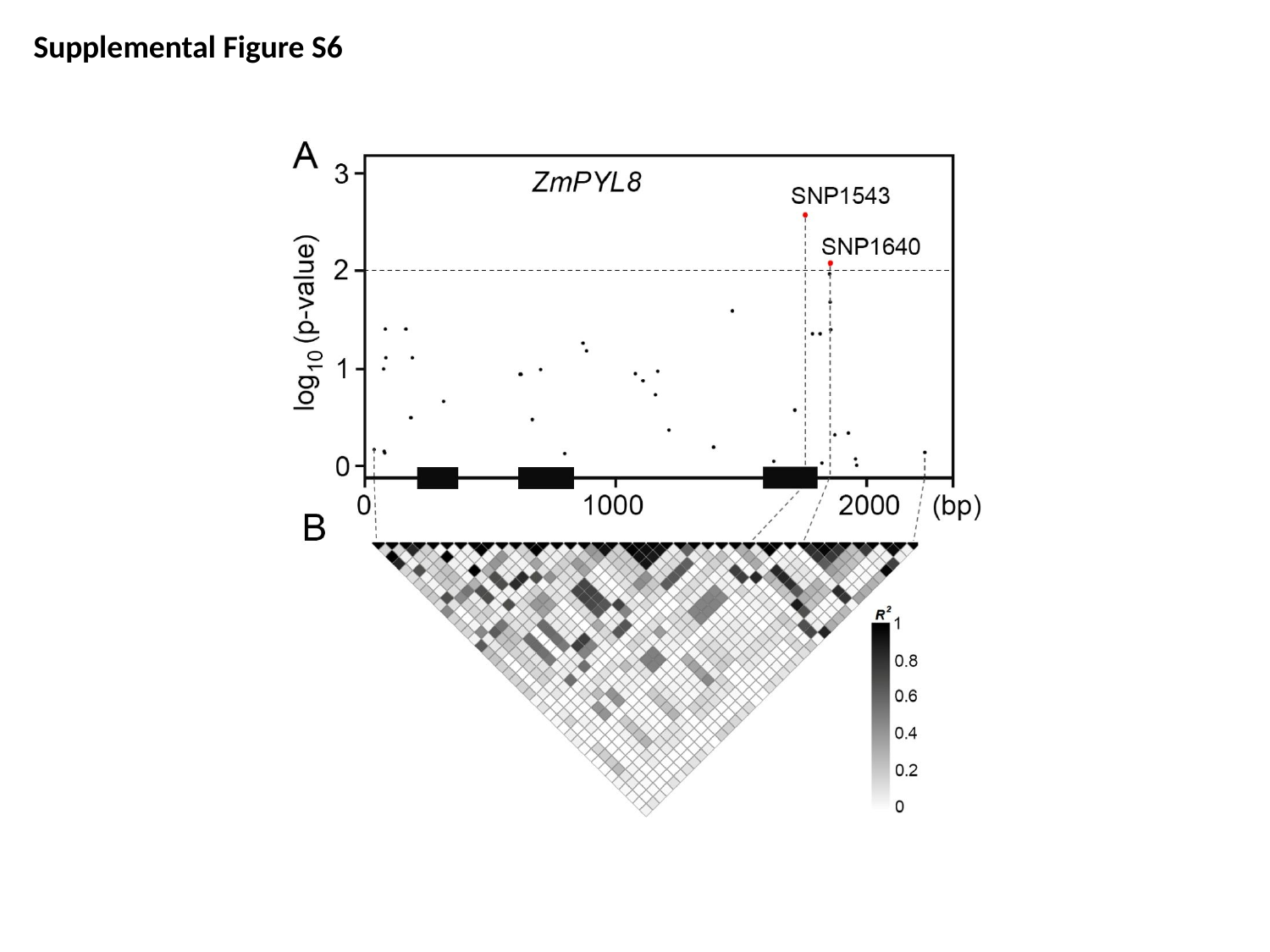

Supplemental Figure S6

## Slide 10
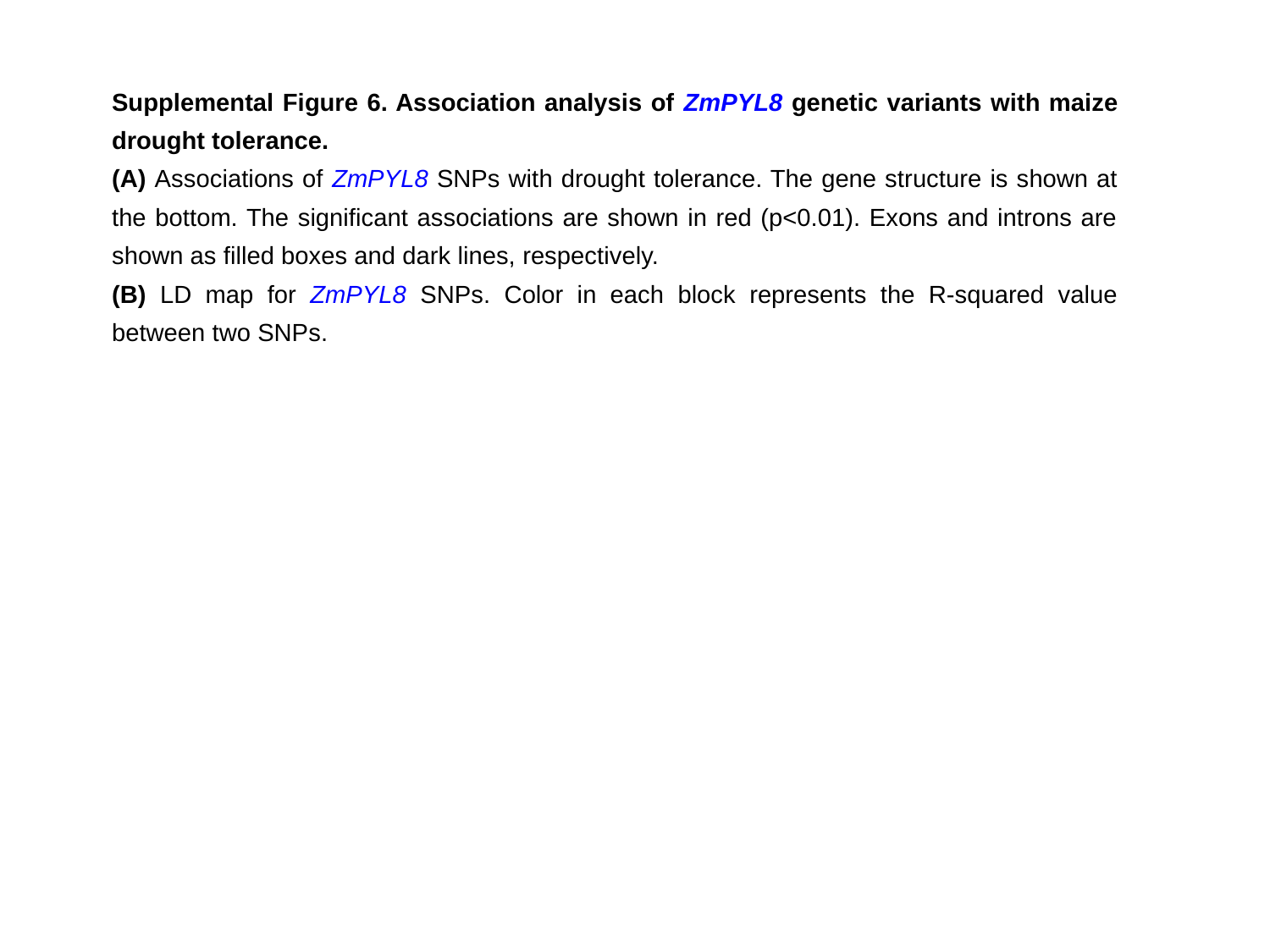

Supplemental Figure 6. Association analysis of ZmPYL8 genetic variants with maize drought tolerance.
(A) Associations of ZmPYL8 SNPs with drought tolerance. The gene structure is shown at the bottom. The significant associations are shown in red (p<0.01). Exons and introns are shown as filled boxes and dark lines, respectively.
(B) LD map for ZmPYL8 SNPs. Color in each block represents the R-squared value between two SNPs.
